# Topography and Radiological Variables as Ancillary Parameters for Evaluating Tissue Adherence, Hypothalamic–Pituitary Dysfunction, and Recurrence in Craniopharyngioma: An Integrated Multidisciplinary Overview

**DOI:** 10.3390/cancers16142532

**Published:** 2024-07-13

**Authors:** Rosalinda Calandrelli, Gabriella D’Apolito, Matia Martucci, Carolina Giordano, Chiara Schiarelli, Giammaria Marziali, Giuseppe Varcasia, Luca Ausili Cefaro, Sabrina Chiloiro, Simone Antonio De Sanctis, Simona Serioli, Francesco Doglietto, Simona Gaudino

**Affiliations:** 1 Department of Imaging, Radiation Therapy and Hematology, Catholic University of the Sacred Heart, Fondazione Policlinico Universitario A. Gemelli IRCCS, 00168 Rome, Italy; gabriella.dapolito@policlinicogemelli.it (G.D.); matia.martucci@policlinicogemelli.it (M.M.); carolina.giordano@guest.policlinicogemelli.it (C.G.); chiara.schiarelli@policlinicogemelli.it (C.S.); giammaria.marziali@policlinicogemelli.it (G.M.); giuseppe.varcasia@guest.policlinicogemelli.it (G.V.); luca.ausilicefaro@policlinicogemelli.it (L.A.C.); simona.gaudino@unicatt.it (S.G.); 2 Pituitary Unit, Division of Endocrinology and Metabolism, Fondazione Policlinico Universitario A. Gemelli, IRCCS, 00168 Rome, Italy; schiloiro@gmail.com (S.C.); simonedesanctiss123@gmail.com (S.A.D.S.); 3 Facoltà di Medicina e Chirurgia, Università Cattolica del Sacro Cuore, 20123 Rome, Italy; francesco.doglietto@unicatt.it; 4 Division of Neurosurgery, Department of Medical and Surgical Specialties, Radiological Sciences and Public Health, Spedali Civili of Brescia, University of Brescia, 25123 Brescia, Italy; s.serioli002@unibs.it; 5 Department of Neurosurgery Fondazione Policlinico Universitario Agostino Gemelli IRCCS, 00168 Rome, Italy

**Keywords:** craniopharyngiomas, MRI, radiological variables, outcomes

## Abstract

**Simple Summary:**

Craniopharyngiomas are benign but complex WHO grade I extra-axial epithelial neoplasms. Their infiltrative nature towards adjacent structures, such as the hypothalamic–pituitary axis, limits surgical excision and increases the rate of intra- and postoperative complications. An accurate depiction of the craniopharyngioma topography, craniopharyngioma–brain interface, and tumor morphology is valuable in the diagnostic work-up of craniopharyngiomas and may address treatment strategies, aiming to balance the risk of postsurgical hypothalamic dysfunction with recurrence rates. The purpose of this pictorial essay is to provide an overview of the role of imaging in both diagnosis and early post-treatment follow-up, highlighting its usefulness in the diagnostic process and its role in outcome prediction.

**Abstract:**

Craniopharyngiomas continue to present a challenge in clinical practice due to their heterogeneity and unpredictable adherence to vital neurovascular structures, particularly the hypothalamus. This results in different degrees of hypothalamus–pituitary axis dysfunction and a lack of uniform consensus and treatment guidelines regarding optimal management. MRI and CT are complementary techniques in the preoperative diagnostic phase, enabling the precise definition of craniopharyngioma size, shape, and consistency, as well as guiding classification into histopathological subtypes and topographical categories. Meanwhile, MRI plays a crucial role in the immediate postoperative period and follow-up stages by identifying treatment-related changes and residual tumors. This pictorial essay aims to provide an overview of the role of imaging in identifying variables indicative of the adherence degree to the hypothalamus, hypothalamic–pituitary dysfunction, the extent of surgical excision, and prognosis. For a more comprehensive assessment, we choose to distinguish the following two scenarios: (1) the initial diagnosis phase, where we primarily discuss the role of radiological variables predictive of adhesions to the surrounding neurovascular structures and axis dysfunction which may influence the choice of surgical resection; (2) the early post-treatment follow-up phase, where we discuss the interpretation of treatment-related changes that impact outcomes.

## 1. Introduction

Craniopharyngiomas (CPs) are benign but complex WHO grade I extra-axial epithelial neoplasms of the sellar and parasellar region [1], representing approximately 2–5% of adult intracranial tumors [2,3] and up to 15% of intracranial tumors in children [4]. They can arise anywhere along the path of the craniopharyngeal duct, but they primarily occur in the suprasellar/sellar area [2]. The vast majority have a suprasellar component, with only around 5% of tumors being solely intrasellar [2].

CP continues to pose a significant challenge in clinical practice. The tumor’s pathobiology remains poorly understood, and there is a lack of consensus and treatment guidelines regarding the optimal management approach for this relatively rare condition [5].

Despite its benign histological appearance, it may cause local invasiveness with an unpredictable growth pattern, affecting critical adjacent anatomical structures such as the brainstem, hypothalamus, pituitary stalk (PS), and optic nerves [6,7]. This may result in the development of hypothalamus–pituitary axis dysfunction, raised intracranial pressure, and visual compromise. For this reason, CPs are often labeled as tumors exhibiting “malignant behavior”, posing challenges in the surgical removal, recurrence rate, and morbidity rate [8,9].

Recently, with the aid of genetic sequencing technology, the World Health Organization (WHO) has classified CPs into the following two distinct categories: adamantinomatous CPs (ACPs) and papillary CPs (PCPs), with unique patterns of age distribution, and genetic and molecular make-ups [10]. ACPs are more common than PCPs and are driven by somatic mutations in CTNNB1 (encoding β- catenin) that affect the Wingless pathway (WNT pathway) [11]; they exhibit a bimodal age distribution, with a peak incidence occurring between the ages of 5–15 and 45–60 [6,12], although they can occur at any age and have even been detected in utero [13]. By contrast, PCPs frequently harbor somatic BRAFV600E mutations which result in the activation of the mitogen-activated protein kinase (MAPK) signaling pathway [14,15]; they are almost exclusive to adults, peaking between the ages of 40 and 55 years [9,16]. To date, tumor molecular characterization has become crucial for assessing the response to targeted therapy and prognosis in craniopharyngiomas [5]. However, while agents that target the WNT pathway remain in development, the availability of BRAF inhibitors such as vemurafenib and dabrafenib has proven effective in reducing the tumor size in patients with papillary craniopharyngiomas [17,18,19].

The differential diagnosis of these two subtypes is crucial because the ACP subtype is associated with higher recurrence rates and poor prognoses compared to PCPs, mainly due to its infiltrative nature causing difficulties in radical resection [20]. However, the variable biological behavior among CP patients, in terms of surgical removal, recurrence rate, and morbidity, is influenced not only by the histological subtype but also by its extreme heterogeneity in terms of consistency, calcification pattern, and topographical categories. These factors altogether affect the degree of adherence to surrounding structures, impacting the surgical planning and outcomes. Understanding preoperatively the type of CP–hypothalamus attachment and the tumor features may provide valuable information. It can help anticipate individual surgical risks, preserve the functional integrity of the attached hypothalamic structures, and predict the risk of tumor recurrence.

Magnetic resonance imaging (MRI) plays a pivotal role in diagnosing and evaluating CPs postsurgery, while computed tomography (CT) imaging serves as an adjunctive method for detecting the presence of fine or coarse calcifications [21,22]. To date, the exact timing for postoperative imaging for CPs is not established, but imaging within two weeks after surgery is commonly suggested to monitor the residual tumor size, assess any signs of recurrence or progression, and evaluate the treatment effects on the surrounding brain structures [23].

This pictorial essay aims to provide a comprehensive overview of the role of imaging in exploring crucial radiological variables indicative of the adherence severity to the wall, axis dysfunction, extent of surgical excision, and prognosis.

Specifically, we delineate the following two distinct scenarios: (1) the initial diagnosis phase, where we primarily discuss the role of radiological variables predictive of adhesions to surrounding neurovascular structures and axis dysfunction, and (2) the early post-treatment follow-up phase, where we discuss the interpretation of treatment-related changes following surgery and/or radiotherapy, which pose a challenge in assessing the presence and extent of residual disease, consequently strongly impacting outcomes.

Finally, we discuss current trends and the utilization of radiomics and artificial intelligence (AI) in the diagnostic process of CPs.

## 2. Methods

### 2.1. Search Strategy

The literature was searched from January 1971 to April 2024 in the medical database PubMed using the following MeSH terms: “Craniopharyngiomas and Topography”, “Craniopharyngiomas and Radiological Variables”, “Craniopharyngiomas and Adherences”, “Craniopharyngiomas and Hypothalamic-Pituitary Dysfunction”, “Craniopharyngiomas and Imaging and Recurrence” “Craniopharyngiomas and Surgical Treatment Strategies”, and "Craniopharyngiomas and Artificial Intelligence”.

### 2.2. Eligibility Criteria

The inclusion criteria were as follows: (1) prospective and/or retrospective observational studies written in English; (2) studies describing a relationship between CPs and clinical–surgical outcomes; (3) reviews, case reports, and case series in adult and child patients. Exclusion criteria included (1) studies with no relationship between CPs and clinical–surgical outcomes, (2) animal studies, (3) and non-English papers.

### 2.3. Study Selection

**Figure 1:**
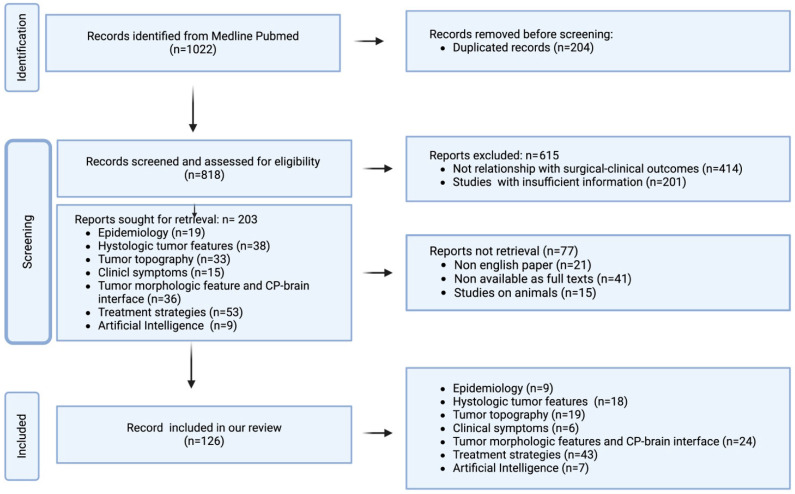
Flow diagram of literature search and article selection. n: number.

The literature revision was performed by three independent authors (two neuroradiologists and one neurosurgeon) and studies were selected by consensus. Duplicates were removed. Titles and abstracts were initially reviewed, and articles that did not meet all of the selection criteria were excluded. Subsequently, the full texts of the included articles were searched, and any unavailable articles were also excluded. The study selection strategy is shown in Figure 1.

### 2.4. Data Extraction

Patients’ data were extracted by 3 authors (R.C., G.D., and F.D.). The extracted data were entered into a database with the following information: epidemiology, histologic tumor features, tumor topography, clinical symptoms, radiological imaging findings, treatment strategies, and outcomes.

## 3. Results

### Medline Review

Although we considered articles from 1971, most of the selected articles were from the last 15 years. The older articles have also been considered because they contained valuable radiological information. We identified 1022 articles concerning predictive variables of adherence and recurrence in patients with CPs. A total of 204 articles were excluded due to duplication, and an additional 615 were excluded because they did not align with the objectives of the review (n = 414) or because they were studies with insufficient information (n = 201). Subsequently, 203 studies were initially selected, with further exclusions made for non-English papers (n = 21), lack of full-text availability (n = 41), and studies involving only animal subjects (n = 15). In total, our review included 126 studies based on epidemiology (n = 9), histologic tumor features (n = 18), topography (n = 19), clinical symptoms (n = 6), morphologic tumor features and peritumoral parenchymal changes (n= 24), treatment strategies (n = 43), and artificial intelligence (n = 7). The results are summarized in Table 1 and Table 2.

## 4. Discussion

### 4.1. Diagnosis Phase

An accurate depiction of the CP topography, CP–brain interface, and tumor morphology is valuable in the diagnostic work-up of CPs. Both MRI and CT are useful techniques for the CP characterization, while, for the tumor site assessment, both neuroimaging and intraoperative evaluation are crucial.

#### 4.1.1. Topographical Classifications

Several CP classifications have been proposed taking into account several CP features, such as the tumor location, direction of growth and extension, degree of hypothalamic anatomical distortion, and the relationships of the tumor to the sellar diaphragm, optic chiasm, PS, and third ventricle (3 V) [39,52,59,60,61,62,63,64,65,66].

Radiological evaluation has been proven as a reliable approach for CP topography. Some radiological methods specifically considered the CP–hypothalamus relationship [24,63,64], whilst other, more accurate, methods assessed the combined relationship among CPs with several anatomical structures of the sellar–parasellar region [52].

In 2017, Prieto et al. [52] proposed the following preoperative MRI variables to accurately define the CP topography: (1) occupation of the 3 V, (2) distortion of the PS, (3) the relative position of the hypothalamus in relation to the tumor, (4) the occupation of the chiasmatic cistern, (5) the angle of the mammillary body (MBA: angle formed by the intersection of the plane tangential to the base of the mammillary bodies with the plane tangential to the floor of the fourth ventricle), (6) the type of chiasm distortion, and (7) the tumor shape. Later studies reported the usefulness of the following three main radiological variables for the correct attribution of the tumor’s origin: (1) the position of the hypothalamus in relation to the tumor, (2) the type of PS distortion, and (3) the tumor shape [39].

Comparison between radiological and intraoperative assessments has shown that tumors with similar radiological features may exhibit peculiar relations and adherences with the surrounding neurovascular structures during surgery, making preoperative radiological planning unpredictable. This apparent mismatch is due to neuroimaging classification systems that reflect the tumor stage at the time of diagnosis and do not always accurately identify the tumor’s point of origin.

The intraoperative evaluation of the relationship between the tumor and the leptomeningeal surface of the third ventricular floor (TVF) has proven as crucial in determining the true epicenter of the tumor’s origin, significantly impacting patient outcomes [67,68,69]. Thus Prieto et al. in 2018 proposed a new and comprehensive intraoperative topographic classification based on the severity of adherence [37].

This classification defined the adherence severity considering the following three components:

(a) The relationship of the tumor with some specific structures (sella, PS, third ventricle floor, and walls);
(b) The tumor’s adhesion morphology, categorized into the following six patterns of attachment: (1) pedicle attachment in the case of gliovascular stem attachment, (2) sessile attachment in the case of patch adhesion, (3) cap-like attachment in the case of wide adhesion involving the upper half of the tumor, (4) ring-like attachment in the case where the center of the tumor surface is attached to a circular band of brain tissue, (5) bowl-like attachment in the case of wide adhesion involving the lower half of the tumor, and (6) circumferential attachment in the case where the entire tumor surface is attached to the surrounding brain tissue;
(c) The adhesion strength, categorized into the following: (1) loose, for easily dissectible adhesion; (2) tight, when sharp tumor dissection is required to preserve the anatomical structure attached; (3) fusion, when a cleavage plane between the tumor and the adjacent brain tissue cannot be identified; (4) replacement, when the structure involved in the attachment is not recognizable.

The combination of these three components determines the following five hierarchical levels of adherence severity with gradually increasing surgical risk of hypothalamic injury [37]:

Level I, or mild, observed in CPs developed below an intact TVF and separated from the hypothalamus by leptomeningeal layers;

Level II, or moderate, for purely intraventricular CPs with a small attachment to the inner lining of the 3 V, which can usually be straightforwardly released;

Level III, or serious, for CPs developed in the suprasellar area whose adhesion to the outer aspect of the infundibulum should be sharply dissected to avoid hypothalamic injury;

Level IV, or severe, in CPs originated within the infundibulum/tuber cinereum showing a wide area of the tumor surface fused to the surrounding remnants of the TVF, which prevents a safe total removal;

Level V, or critical, for CPs attached to the whole pituitary–hypothalamic axis that have invaded and replaced the TVF.

Prieto et al. demonstrated that the CP adherence to adjacent tissues and structures, based on the relationship between the tumor and the leptomeningeal surface of the third ventricular floor, provided a basis for an objective classification of CPs in the three main topography categories. For each topography category, they related intraoperative data with the main neuroradiological predictive variables detected in previous studies [39,52].

Below, we arranged the most significant intraoperative findings and the associated preoperative radiological variables capable of predicting each CP topography.

**Figure 2:**
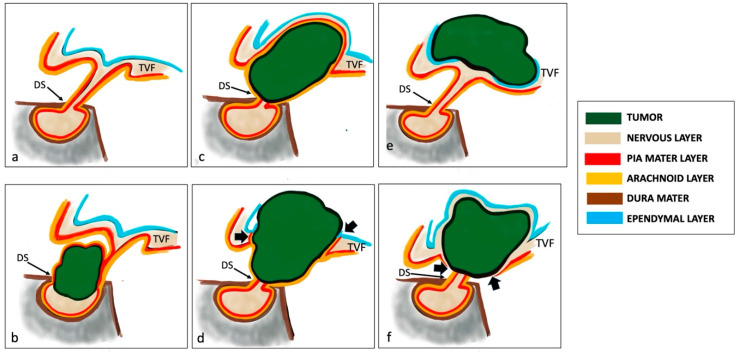
Relationship between tumor and leptomeningeal surface of the third ventricular floor in craniopharyngiomas. Normal representation of pituitary gland and meningeal layers (**a**). Dura mater layer is illustrated in brown; pia mater layer is shown in red; arachnoid layer is shown in orange; nervous layer is shown in light brown; ependymal layer is represented in blue. The TVF is covered from the ventricular ependymal layer. Sellar–suprasellar category. Sellar–suprasellar CP in (**b**). The CP arises below the sellar diaphragm but grows within the infrasellar and/or intrasellar cavity. It is separated from the TVF by the leptomeningeal layer (arachnoid and pia mater). Suprasellar category. (1) Pseudo-third ventricle CP in (**c**). It originates in the suprasellar subarachnoid spaces and exhibits a cap-like adhesion to the outer leptomeningeal surface of the TVF. The arachnoid layer typically wraps around the tumor. (2) Secondary intraventricular CP in (**d**). The CP originates in the suprasellar subarachnoid spaces. Initially, it develops beneath the 3 V but, in late stages of development, it extends into the 3 V after breaking through the TVF (black arrows) and becomes intraventricular. It has attachment to the pial surface of the infundibulum and/or tuber cinereum, without an intervening arachnoid layer. Intraventricular category. (1) Strictly intraventricular CP in (**e**). The tumor is located above an intact TVF and protrudes into the ventricular cavity without an ependymal cellular layer or an identifiable neural tissue-covering layer. Not-strictly intraventricular CP in (**f**). It is considered a truly intra-hypothalamic lesion. It is situated within the ventricular floor, featuring an ependymal cellular layer and a nervous layer covering the tumor’s surface. The leptomeningeal surface of the TVF is interrupted (black arrows). CPs, craniopharyngiomas; TVF, third ventricular floor; 3 V, third ventricle; DS, dorsum sellae.

**Figure 3:**
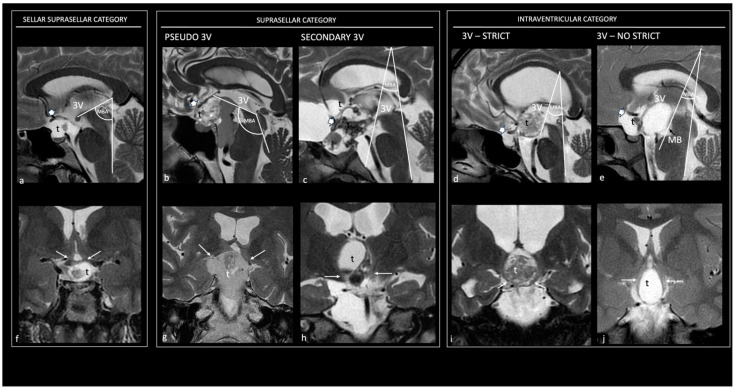
Topographical categories of craniopharyngiomas. Preoperative sagittal (**a**–**e**) and coronal (**f**–**j**) T2 magnetic resonance images show the five major topographical categories of craniopharyngiomas. Sellar–suprasellar category. Sellar–suprasellar CP (**a**–**f**): the chiasmatic cistern is occupied by the tumor, the PS is not visible, the chiasm is stretched upward (thick white arrow in (**a**)), the mammillary body angle (MBA) is acute (65°), and the hypothalamus (thin white arrows in (**f**)) is around the upper-third level of the tumor; the 3 V is free of tumor. Suprasellar category. Pseudo-third ventricle CP (**b**,**g**): the sella, chiasm cistern, and 3 V are occupied by a giant tumor; the PS is not visible, the chiasm is stretched upward (thick white arrow in (**b**)), the mammillary body angle is obtuse (120°), and the hypothalamus (thin white arrows in (**g**)) is around the upper third of the tumor. Secondary intraventricular CP (**c**,**h**): the chiasm cistern and the 3 V are occupied by the tumor, the PS is not visible, the chiasm is stretched forward (thick white arrow in (**c**)), the angle of the mammillary body is acute (40°), and the hypothalamus is located around the mid-third of the tumor (thin white arrows in (**h**)). Intraventricular category. Strictly intraventricular CPs (**d**,**i**): the chiasm cistern is tumor-free, the PS is entirely visible, the chiasm is compressed downward (thick white arrow in (**d**)), the angle of the mammillary body is acute (35°), and the hypothalamus is in the lower-middle third of the tumor (thin white arrows in (**h**)). Not-strictly intraventricular CPs (**e**,**j**): the chiasm cistern is occupied by tumor, the chiasm is compressed forward (thick white arrow in e), the angle of the mammillary body is hyperacute (30°), and the hypothalamus is located around the mid-third of the tumor (thin white arrows in (**j**)). 3 V, third ventricle; Ch, chiasm; MBA, mammillary body angle; t, tumor.

**1. Sellar–suprasellar category.** This encompasses CPs originating below the sellar diaphragm, growing within the infrasellar and/or intrasellar cavity, and being separated from the TVF by a layer of leptomeningeal tissue (arachnoid and pia) (Figure 2b). They display a sessile pattern of attachment and have easily dissectible adhesions (Level I of adherence severity) [39]. The hypothalamic position around the upper-third level of the CF, a non-visible PS, a wholly occupied chiasmatic cistern, an MBA ranging between 60° and 89°, an upwardly stretched chiasm, and a pear-shaped or, less commonly, dumb-bell-shaped tumor are the preoperative radiological variables suggesting membership in this topography [37,52] (Figure 3a,f).

**2. Suprasellar category.** This comprises primarily extraventricular tumors originating above the diaphragm sellae and developing from the suprasellar segment of the PS. Within this category, a sharp distinction should be made between those lesions that push upwards on an intact TVF (pseudointraventricular CPs) and those that have invaded the 3 V after breaking through the TVF (secondarily intraventricular CPs) [70]. Suprasellar–pseudointraventricular CPs are extraventricular tumors but mimic an intraventricular position. They originate in the suprasellar subarachnoid spaces and exhibit a cap-like adhesion to the outer leptomeningeal surface of the third ventricular floor. The arachnoid layer typically wraps around the tumor; this meningeal layer interposed between the tumor and the TVF makes this tumor category easily dissectible (Level I of adherence severity) [39] (Figure 2c). Their development may be either prestalk or retrostalk, depending on the orientation of tumor expansion. The hypothalamic position around the upper-third level of the CP, a non-visible PS, a wholly occupied chiasmatic cistern, an MBA greater than 90°, an upwardly stretched chiasm, and a multilobulated or, more rarely, pear-like tumor shape are the preoperative radiological variables able to predict belonging to this topographic class [37,52] (Figure 3b,g). Secondary intraventricular CPs initially develop beneath the 3 V; however, in the late stages of development, they extend into the 3 V after breaking through the TVF, thus becoming intraventricular. They originate in the suprasellar subarachnoid spaces but may exhibit serious, severe, or critical adherences (Levels III, IV, and V of adherence severity) due to their potential ring-like attachment to the pial surface of the infundibulum and/or tuber cinereum, without an intervening arachnoid layer, and typically replacing the TVF with the tumor mass [39] (Figure 2d). The hypothalamic position around the central portion, a non-visible PS, a wholly occupied chiasmatic cistern, an MBA ranging between 30° and 59°, a chiasm stretched forward, and a multilobulated tumor shape are the preoperative radiological variables that suggest belonging to this topographic class [37,52] (Figure 3c,h).

**3. Intraventricular category (IVCs).** This category of CPs grows largely or wholly within the 3 V (strictly and not-strictly intraventricular CPs), originating above the leptomeningeal layer and being subependymal. They are situated around the infundibulo-tuberal region, typically above a patent suprasellar cistern. A clear topographical assignment of these two subtypes may not always be definitively determined even intraoperatively. When differentiated, strictly CPs are masses located above an intact TVF, widely protruding into the 3 V cavity, without an ependymal cellular layer or an identifiable neural tissue-covering layer [71] (Figure 2e). They exhibit a narrow attachment via a pedunculated, vascular–gliotic stalk to the antero-inferior area of the TVF, demonstrating moderate adherence to the TVF (Level II of adherence severity) [39,72]. Radiological clues strongly indicative of this category include the hypothalamic position around the lower third of the tumor, the presence of a tumor-free chiasmatic cistern, an intact PS, an MBA between 30° and 59°, a downwardly compressed chiasm, and a round tumor shape [37,52] (Figure 3d,i). Conversely, not-strictly CPs can be considered as truly intra-hypothalamic lesions. They are situated within the ventricular floor, featuring an ependymal cellular layer and a nervous layer covering the tumor’s surface [71] (Figure 2). These tumors are distinguished by the most wide and strong adhesions to the hypothalamus, characterized by the fusion between the neural tissue and the central-lower portion of the lesion (bowl-like), or the entire surface (circumferential) of the CP capsule in more than two-thirds of cases, demonstrating severe or critical adherence to the TVF (Levels IV and V of adherence severity) [39]. The hypothalamic position around the central portion of the tumor, an amputated and, in some cases, infiltrated PS, a partially occupied chiasmatic cistern, an MBA less than 30°, a downward or forward compressed chiasm, and an elliptical tumor shape are the presurgical radiological variables indicating belonging to this topographic class [37,52] (Figure 3e,j).

Some authors have explored the relationship between CP topographies and histological subtypes (ACPs and PCPs) and they found that the squamous-papillary histological pattern was predominantly associated with strictly intraventricular CPs, while the adamantinomatous pattern was predominantly observed in the other topographical categories [24].

#### 4.1.2. CP–Brain Interface

A very limited number of studies reported on the CP–brain interface. Parenchymal peritumoral changes induced by CPs on the surrounding nervous structures, attributable to edematous phenomena or gliosis, have been identified in less than half of the tumors [38,73]. These perifocal changes are more frequent in some topographic classes and in the ACP subtype, but depend mainly on the tumor’s growth pattern and its distorting effects on the nearby hypothalamic region [70]; in fact, peritumoral changes may vary even among tumors belonging to the same topographic class and within the same histopathological subtype [54].

The presence of a thick layer of reactive gliosis at the CP–brain interface has been demonstrated to represent an indirect marker of rapid tumor growth and invasion/infiltration into the adjacent brain tissue [20,34,67,69,74]. It is never observed in sellar/suprasellar CPs and is not usually observed in strictly intraventricular CPs, while it is frequently present in the suprasellar CP category and very often observed in the infundibulo-tuberal topography, where tumors frequently exhibit finger-like epithelial projections into the adjacent brain [37,70]. In infundibulo-tuberal CPs, the thick layer of gliosis is reported to represent a primary reaction of the hypothalamic area in contact with the original site of the CP [75,76]; conversely, in tumors pushing against the TVF (pseudointraventricular tumors) or those that have invaded the 3 V (secondary intraventricular cases), a gliotic reaction is likely due to ependymal disruption and lymphocytic infiltration of the 3 V walls or to the mechanical pressure exerted by the tumor on the adjacent neural tissue [60,71,77].

Inflammatory reaction of the adjacent brain tissue, prevalently along the 3 V walls, has been described mainly in cystic CPs. It is induced by the chronic leakage of the highly irritating lipid-rich oily content from the cyst, derived from desquamated epithelial cells [37,38,50,78].

Parenchymal perifocal edemas induced by CPs have been predominantly observed along the visual pathways. Some authors consider it a sign of CP invasiveness/infiltration [79], while others attribute it to the distension of the large perivascular spaces (Virchow–Robin spaces), which are consistently present along the middle portion of the optic tracts and typically communicate with the adjacent subarachnoid space [80]. In the latter case, the edema may represent a reaction to the CP itself secondary to a focal disturbance in the cerebrospinal fluid (CSF) [80].

#### 4.1.3. Morphologic Tumor Features

CPs usually appear as heterogeneous tumors on imaging; they are described as purely or predominantly cystic in 46–64% of cases, purely or predominantly solid in 18–39% of cases, and mixed in 8–36% of cases [81]. Their MRI appearance depends on the proportion of solid and cystic components, the content of the cyst(s) (cholesterol, keratin, and hemorrhage), and the amount of calcification present [30]. Large calcifications can be seen in MRI studies, while fine calcifications are difficult to detect in MRI studies and require the use of CT [28,30].

No significant differentiating radiologic characteristics have been established for CPs in children versus adults [82]. Moreover, imaging cannot always accurately differentiate ACPs from PCPs, especially in adult patients, where variations in morphological characteristics are not uncommon [28]. In the most typical cases, some radiological clues such as tumor shape, composition, location, and the enhancement pattern may be helpful in determining the histotype.

**Figure 4:**
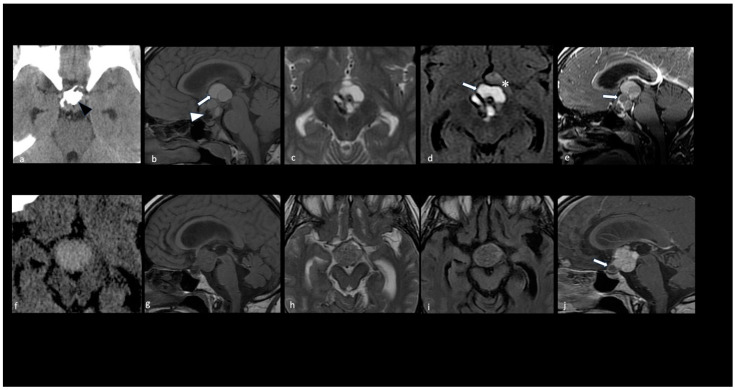
Key morphologic features in adamantinomatous and papillary craniopharyngiomas. CT scan (**a**,**f**); sagittal T1 (**b**,**g**), axial T2 (**c**,**h**), axial FLAIR (**d**,**i**), and sagittal T1 with contrast medium (**e**,**j**) MRI. Adamantinomatous craniopharyngioma (**a**–**e**). Note a predominantly cystic multilobulated suprasellar mass with an intratumoral coarse calcification pattern (black arrowhead in (**a**)). The MRI shows the following: T1 shortening and FLAIR/T2 hyperintensity within the multicystic tumor component due to machine oil-like proteinaceous fluid (white arrows in (**b**,**d**)); T1 hypointense elements representing calcifications (white arrowhead in (**b**)); rim enhancement of the wall of the cysts on T1 with a contrast medium (white arrow in (**e**)). Parenchymal perifocal edema-like changes along the left optic tract and chiasm are shown in (**d**) (*). Papillary craniopharyngioma (**f**–**j**). Note a predominantly solid suprasellar mass without evidence of calcifications. The solid component appears hyperdense on the CT (**f**) with a homogeneous enhancement; the small cystic component is caudal and shows the rim enhancement of the wall (white arrow in (**j**)).

ACPs commonly have a lobulated, multiloculated appearance, containing both cystic and solid components as well as calcifications [26,33,34,35,36]. The cystic component is usually prevalent and contains fluid with the appearance of ‘machinery oil’ or a ‘shimmering’ quality, attributed to the high cholesterol content resulting from desquamated squamous epithelial cells rich in membrane lipids and cytoskeleton keratin [3]; however, a hematic-like cystic pattern and CSF-like cystic pattern may coexist [31]. The cyst wall frequently exhibits contrast enhancement and eggshell-like calcifications [28,29,34]. The solid component, representing a minor part of the lesion, usually exhibits reticular contrast enhancement [30] (Figure 4a–e).

Conversely, PCPs exhibit a spherical appearance, can be entirely solid or a mixture of cystic and solid components, and do not typically calcify [25,26,32]. In cases where a cystic component is present, the content of the cyst is usually clear or yellow, often resembling a CSF-like pattern, while a viscous content is rarely observed [2,25,32] (Table 1, Figure 4f–j).

#### 4.1.4. Differential Diagnosis

Although neuroradiology is useful for CP assessment, differential diagnosis may be a challenge when its radiological features overlap and resemble other lesions of the sellar and parasellar region.

In cases of purely cystic CPs, the main diagnostic challenges—both in children and adults—are Rathke’s cleft cysts (RCCs), arachnoid cysts, dermoid cysts, and epidermoid cysts [83]. However, the following peculiar findings can guide the final diagnosis: (1) the presence of a non-enhancing intracystic proteinaceous nodule with a low signal intensity on T2-weighted images, the absence of wall enhancement [84], and the absence of calcification are indicative of an RCC [85,86]; (2) a CSF-like lesion lacking calcification and wall enhancement, which favors an arachnoid cyst [83]; (3) a cystic lesion exhibiting a high signal on T1 images that is suppressed on fat-saturation sequences (due to lipid content), the presence of the foci of calcifications, and no wall enhancement are indicative of a dermoid cyst [87]; (4) a CSF-like cystic lesion with restricted diffusion is a diagnostic hallmark of an epidermoid cyst [88].

In cases of solid or mixed solid–cystic CPs, age should be considered in the diagnostic assessment.

In the pediatric population, the main differential diagnoses are hypothalamic–optic gliomas (HOGs), germ cell tumors (GCTs), and, less frequently, histiocytic tumors such as juvenile xanthogranuloma and Langerhans cell histiocytosis. A lesion with a very hyperintense T2 signal and the absence of calcifications, along with the physiological T1 hyperintense signal of the posterior pituitary gland, favors a HOG [89,90]; the restricted diffusion of the solid component of the lesion, reflecting hypercellularity, and infundibular thickening are diagnostic hallmarks of GCTs [91,92]; a mass with an infiltrative pattern, layering along the PS, indicates Langerhans cell histiocytosis [93].

In adults, the main differential diagnoses are PitNET, tuberculum sellae meningioma, and thrombosed aneurysms. Infrasellar tumor extension with sellar expansion, the inability to separately identify the pituitary gland from the mass, and the absence of calcifications are distinctive signs of PitNET [94]; the meningeal tail sign is pathognomonic of meningioma [2]; an eccentric pattern of concentric circles within a mass located adjacent to arterial vascular structures (such as the anterior cerebral artery and internal carotid artery) suggests a thrombosed aneurysm [29].

#### 4.1.5. Preoperative Variables Predictive of the Severity of CP Adherence

Several imaging studies on CPs have aimed to identify preoperative radiologic features that could be used to predict the severity of tumor adherence to critical surrounding structures, the subtype, and, more recently, the mutation status [24,39,63].

To date, there is overall agreement that CP topography is the major determinant of the degree of adherence to surrounding structures, reflecting the difficulty of surgical removal [39].

The least severe type of adherence (Level I) was described in sellar–suprasellar and suprasellar–pseudointraventricular topographies, the slightly higher type of adherence (Level II) was observed in strictly intraventricular CPs, while the next levels of adherence severity (Levels III–V) were described in secondary intraventricular (Levels III–V) and not-strictly intraventricular CPs (Levels IV and V) [39]. 

Several radiological studies have demonstrated that the degree of hypothalamic distortion, assessed using various methods, is the primary radiological variable that influences the severity of tumor adherence to the surrounding brain tissue [24,63,64]. Subsequently, other radiological studies combining hypothalamic distortion with additional radiological variables have demonstrated that the position of the hypothalamus in relation to the tumor, along with the type of PS distortion and the tumor shape, could predict, with high diagnostic accuracy, the severity of tumor adherence to the surrounding brain tissue, suggesting the belonging to a particular topographic category. The position of the hypothalamus around the mid-third portion of the tumor, signs of an amputated or infiltrated stalk by the lesion, and elliptical or multilobulated tumoral shapes are strong predictors of the infundibulo-tuberal and secondarily intraventricular topographies, which are characterized by strong and extensive CP adhesions to the hypothalamus [39].

Also, the presence of gliotic, edema, and inflammatory changes has been demonstrated to be suggestive of strong adhesion to the hypothalamus, and they are frequently detected in the infundibulo-tuberal topography, where the tumor originates above the leptomeningeal layer and has direct contact with the neural tissue of the TVF [37,38,50,78]. Gliotic/edema-like changes have been demonstrated on T2-weighted MRI axial scans as hyperintense areas in the hypothalamic region or along optic tracts, while local inflammatory reactions have been typically detected as T2 hyperintensity with contrast enhancement along the 3 V walls [38].

The contribution of other factors, such as size and consistency, play a much less important role in CP adhesion patterns [70]. Large cystic tumors that display finger-shaped protrusions into adjacent nervous tissue are independent predisposing factors for forming strong connections to surrounding neurovascular structures, particularly when the capsule is thick or calcified and the cyst contains viscous colloid [27,34,35,36,40,41,42,43,44,45,46,47,48,49] (Table 2).

### 4.2. Clinical Presentation

CPs are often associated with hypothalamic–pituitary dysfunction, and may cause neurologic and endocrinologic complications. It is diagnosed in around 35% of children and adults with CPs just at the time of diagnosis, increasing to 65–80% of cases during disease-related treatments [95].

The hypothalamic–pituitary dysfunction is characterized by the occurrence of hypopituitarism, vasopressin deficit (AVP-D), loss of the thirst reflex, electrolyte disturbances, hypothalamic obesity, and disturbances in the sleep–wake rhythm [95]. The electrolyte disturbances are characterized by sodium imbalance, often with blood hyperosmolar syndrome, involving around 27% of cases [96]. Because of the involvement of the hypothalamic nervous centers, hypothalamic obesity has been reported to be due to both an increase in parasympathetic stimulation, which causes a rapid increase in insulin production and an increase in lipogenesis in the liver and adipose tissue, and the concomitant downregulation of sympathetic tone, which contributes to triglyceride accumulation [97].

Furthermore, because of the essential role of the hypothalamus in the complex neurophysiological process of sleep, CPs may also be responsible for disturbances in sleep–wake regulation, including alterations in circadian rhythm, sleep fragmentation, and increased daytime sleepiness [98].

CP topography along with reactive parenchymal changes at the CP–brain interface are the main variables influencing the functional state of the hypothalamic–pituitary axis [37]. In this regard, hypothalamic dysfunction, which may manifest with electrolyte disturbances, hypothalamic obesity, and disturbances in sleep–wake rhythm, is commonly observed in CPs invading the TVF (secondary intraventricular CPs) and in CPs developing within the TVF itself (both not-strictly intraventricular and strictly intraventricular CPs). On the other hand, pituitary dysfunction, which manifests as panhypopituitarism or anterior hypopituitarism (partial or complete), or diabetes insipidus, is more common in CPs originating in the region of the PS with or without secondary invasion of the 3 V (sellar–suprasellar, pseudointraventricular, and secondary intraventricular CPs) [24,37,54]. The extent of peritumoral edema-like changes also contributes to the severity of hypothalamic dysfunction; it has been reported that more extensive parenchymal edema-like changes are associated with more severe hypothalamic–pituitary dysfunction [54].

### 4.3. Treatment Strategies

Surgical resection is the first choice for most adult-onset CPs [28]. The surgical approach and technique depend on several factors, including the tumor’s size, consistency, location, degree of extension into neighboring structures, and the experience and preference of the surgical team [30]. In the modern era, surgery via the trans-sphenoidal route is generally considered the most favorable approach when possible, yielding better outcomes in terms of the extent of resection, functional sequelae, and complication rates [99,100,101].

The main purpose of CP surgery is gross total resection (GTR), as it is associated with better outcomes [102]. However, the amount of tumor clearance, whether via GTR or subtotal/partial resection followed by adjuvant radiotherapy, remains controversial. This depends on the rate of hypothalamic adherence, which significantly increases the risk of intra- and postoperative complications, limiting the safe radical removal of the tumor [28].

Although the optimal treatment for CP remains controversial, there is consensus that surgical treatment should be tailored to tumor localization, age, and tumor consistency [28,64]. A careful radiological analysis of severe hypothalamic involvement is crucial to establish the most appropriate surgical strategy and discuss the intraoperative challenges with the patient and caregiver [103,104]. Thus, although different therapeutic options are recommended, the treatment should be personalized. Generally, if there is a clear separation between the tumor and the hypothalamus, attempting GTR during the first surgery is considered optimal, as subsequent surgical attempts can increase the mortality and morbidity [103]. Conversely, for tumors with hypothalamic invagination or signs of adherence, subtotal resection with adjuvant radiotherapy is recommended due to the higher risk of hypothalamic damage and negative long-term sequelae [102,103].

In the management of cystic CPs, localized intracystic treatments are most commonly reserved for children and young adults with CPs, where delaying surgery or radiation therapy may be helpful in reducing the risk of complications associated with hypothalamic–pituitary axis dysfunction [30]. In fact, younger patients have been reported to suffer from more chronic, life-altering symptoms than older patients [105]. The insertion of an Ommaya reservoir—an intraventricular catheter accessed subcutaneously—provides an accessible route for recurrent aspiration, as well as the administration of intracystic chemotherapy, radionuclides, or biological therapy [106].

Radiation therapy is an integral part of treating CPs, especially for patients with residual or recurrent tumors who have undergone resective surgery [107,108]. Reports in the pediatric literature have confirmed that the overall survival and progression-free survival in patients who have undergone GTR are similar to those in patients treated with the combination of STR and radiation therapy [109]. Intensity-Modulated Radiation Therapy (IMRT), Fractionated Stereotactic Radiotherapy (FSRT), Stereotactic Radiosurgery (SRS), and proton beam therapy are newer modalities increasingly used in the management of CPs. The choice of modality is largely influenced by the tumor size, as well as the proximity to nearby vital structures [28].

In patients undergoing reoperation for tumor recurrence, the chance of surgical excision is similar to the first surgery, but only for those who did not undergo radiotherapy. The postoperative complication rate is not increased, except for diabetes insipidus [110,111].

Recently, genomic analyses of CP, including mutation, methylation, and transcriptome analyses, have provided considerable insights into its pathophysiology [112]. This finding raised the possibility of targeted therapy with BRAF inhibitors in recurrent or histologically proven PCPs with the BRAF V600E mutation, showing promising results in tumor volume reduction and tumor control [17,113,114]. However, the majority of data available are obtained from single cases or small groups of patients (phase II trial study) [113], so further studies are necessary to better understand the potential of these agents.

### 4.4. Postoperative Phase

#### 4.4.1. Role of Early Postoperative MRI

Early postoperative imaging for CPs is crucial for assessing the presence and extent of residual disease. It also helps to identify potential complications arising from surgical maneuvers due to the tenacity of tumor adhesions to the surrounding tissues or from radiation therapy [23].

To date, the exact timing for postoperative MRI for CPs is not established, but imaging within two weeks after tumor resection is commonly suggested; baseline imaging for response evaluation during later follow-ups should be considered. The extra-axial location of the CP permits a longer interval for postoperative imaging compared to intra-axial tumors, as confounding effects related to non-tumoral marginal enhancement from surgical traumatic and inflammatory processes are not expected [115]. Particular attention should be paid when interpreting the small contrast-enhanced areas adhering to the hypothalamus. They could represent regions of blood–brain barrier disruption secondary to the surgical manipulation of the affected area, complicated by tumor local adhesions [23] or small remnants of the cyst wall capsule [70,116,117]. It has been reported that the recurrence rate is much higher in the case of cystic tumor removal compared to solid tumors; this seems to be explained by the evidence that the cyst wall of CPs contains more tumor stem cell niches, and small remnants may also lead to tumor regrowth [118]. Moreover, fine calcifications at the site of the complete tumor removal should not be underestimated, but rather should raise suspicion of a small residual tumor. Although CT scans are not routinely performed, especially in pediatric patients to minimize radiation exposure, the guidelines for imaging in a previous CP study recommend a postoperative, unenhanced CT of only the tumor region [119]. In this manner, CT scans could reveal persisting calcifications that the postoperative MRI failed to detect, potentially indicating an “early” small residual tumor.

As a general rule, the availability of a preoperative imaging of the CP is essential for accurately assessing postsurgical residual disease. Similarly, knowing the precise surgical approach and any intraoperative resection issues is important for correctly interpreting early MRI findings and later sequelae.

Treatment-related complications, although rare, can be secondary to surgery and/or radiotherapy. Surgery can cause the edema of the hypothalamic region, scarring, and adhesions, as well as optic pathway lesions, injury to the pituitary gland, CSF leakage, hemorrhage, hydrocephalus, and vascular injury including pseudoaneurysms [2,23,120].

Transient signal changes like edema and blood–brain barrier damage have been reported secondary to the manipulation of the brain, and only prolonged clinic–radiologic follow-up imaging may discriminate between them and a permanent lesion in the optic pathways or pituitary gland/PS [23,121].

Radiotherapy may cause the edema of the hypothalamic region, injury to the optic nerve or chiasm, temporal lobe necrosis, and hydrocephalus, the latter likely attributed to inflammation of the epithelial lining of the ventricles. Moreover, in cases of residual tumor, radiotherapy may cause the transient enlargement of cystic or solid components due to changes in the osmotic gradient and permeability of the cyst wall, or due to local inflammation [122,123,124]. Only following studies performed 2–3 months after surgery may help detect true tumor growth and discriminate between transient signal changes and permanent lesions [23].

To date, the severity of parenchymal changes in the hypothalamus region has been suggested as the major predictor of complications which translates to hypothalamic–pituitary dysfunction. MRI plays a key role in categorizing the severity of the damage [38,78]. The extent of hypothalamic involvement has been assessed by imaging using various classification systems proposed by Sainte-Rose and Puget, Van Gompel, and Muller [63,64,78,125]. However, in the postoperative setting, these systems have proven to be inadequate in predicting hypothalamic dysfunction [126]. Hence, some authors have introduced a novel grading system as an index of postoperative hypothalamic dysfunction. This system evaluates the extent of peritumoral edema by categorizing it into the following three classes: class A, absence of edema; class B, edema restricted to the nearby area of the tumor in the hypothalamus; class C, edema extending towards the internal capsule and the optic tract [126].

More extensive parenchymal edema-like changes are associated with more severe hypothalamic–pituitary dysfunction [54].

However, none of the above classification systems distinguish between local edema secondary to surgery and infiltration of adjacent brain tissue [78,80,126].

#### 4.4.2. Recurrence and Radiological Variables Predictive of Recurrence

Longitudinal imaging after a baseline study is necessary to identify tumor recurrence of GTR or growth of partially resected CPs [23]. The timing of follow-up imaging varies, typically involving more frequent scans during the first year after surgery, approximately every 3 months while the patient is on therapy. This is followed by an annual scan if the patient remains stable, or at shorter intervals in case of tumor regrowth [127].

Tumor recurrence occurs in 20–40% of cases and is the most important factor in determining survival. The recurrence rate mainly reflects the surgical result achieved. Radical surgical removal and the use of radiation therapy (RT) after subtotal removal have been recognized as therapeutic factors that significantly decrease the rate of CP recurrence [46,58,128,129,130,131,132]. Nevertheless, total resection does not preclude the recurrence of the tumor [133]; this is probably linked to the concomitant role of certain non-surgical risk factors.

To date, controversy exists regarding the influence of histopathological (subtype, brain invasion) or epidemiological risk factors (age, gender) as predictors of recurrence and clinical outcome in patients with CPs [55,82,134,135]. Contrary to the traditional concept that the adamantinomatous subtype represents a more aggressive lesion than the squamous papillary CP variant [20,57], recent studies have not found significant differences in the recurrence rates between the two histologic subtypes [34]. Moreover, although some studies reported a higher incidence of recurrence in males [134] and in children [45], likely due to delayed or non-utilization of radiation therapy, many other series have not found any correlation between these epidemiologic factors and CP recurrence [82,135].

On the other hand, all investigators agree that CP topography is the best predictor of recurrence.

Specifically, the presence of tight tumor adherence to surrounding neurovascular structures and the loss of the peritumoral gliotic layer, which typically separates the CP from the surrounding hypothalamus, as frequently observed in the group of infundibulo-tuberal CPs, significantly increases the risk of recurrence [27,46,47,51,52,57,58].

Regarding the tumor’s intrinsic morphological features, higher recurrence risk is usually associated with larger cystic CPs [27,34,40,41,42,43,44,45,46,47,48] compared to largely solid CPs [27,40,53]. This increased risk is due to the difficulty in completely removing the cyst wall from the surrounding structures, especially when the capsule is thick or calcified and the cyst contains viscous colloid [40,46,54,55,56] (Table 2).

### 4.5. Radiomics in Craniopharyngioma

Radiomics represents an innovative development in quantitative bioimaging analysis, based on features extraction from standard radiological images such as computed tomography (CT), magnetic resonance imaging (MRI), and positron emission tomography (PET), and selected features may contribute to the performance of radiomics models [136]. Moreover, radiomic features can be analyzed with machine learning (ML) and deep learning (DL) techniques, subfields of artificial intelligence (AI), leading to improved diagnostic performance in clinical oncology [137]. The application of AI in the field of medical imaging, using ML and DP techniques, shortens the image processing time and improves the reliability of diagnostic results by leveraging big data. The applicability and utility of radiomics have already been validated in several tumor types such as gliomas and meningiomas, providing valuable information on their intratumoral heterogeneity [138]. Although some attempts have emerged in recent years in the field of the intelligent diagnosis of craniopharyngioma, research on radiomics and artificial intelligence for diagnosing this condition is still in its preliminary stages. Current research on intelligent diagnosis methods focuses on predicting preoperative craniopharyngioma invasiveness, CTNNB1 and BRAF mutations, and postoperative prognosis. These aspects are crucial for assessing the response to targeted therapy, planning the customization of surgical schemes, and addressing the management of patients during the follow-up [139,140,141,142,143].

MRI-based radiomics methods use different combinations of selected radiomic features extracted primarily from T1-weighted images after the contrast MRI (such as tumor location, intensity, shape, texture and wavelet features, and enhancement patterns) to build radiomics models, as well as supervised ML models.

Chen et al. discussed the prediction of BRAF and CTNNB1 mutations through a radiomics method based on MRI. By incorporating specific radiomics features and using the Random Forest algorithm, they built a radiomics-based model to accurately identify CTNNB1 mutations in ACP tumors [140]. Yue et al. proposed an ML model for discriminating between BRAF-mutated tumors and wild-type tumors, achieving a high sensitivity and specificity [141]. Huang et al. investigated an ML-based multiparametric MRI radiomic model using three methods, including SelectKBest, the least absolute shrinkage and selection operator (LASSO) algorithm, and the support vector machine (SVM), to select the optimal radiomic features from axial T1-weighted (T1-w), T2-weighted (T2-w), and contrast-enhanced T1-weighted (CET1-w) images. The model has been proven effective in distinguishing the two pathological subtypes of CP [139].

Yuen Teng et al. developed convolutional neural network (CNN) models, which are deep learning technologies, to automatically distinguish ACPs from PCPs. Six models were established using six networks, including VGG16, ResNet18, ResNet50, ResNet101, DenseNet121, and DenseNet169. The ResNet50-based model was identified as the optimal architecture in differentiating ACPs from PCPs [142]. In all of these radiomics and ML models, textural features have been proven to be the most effective for estimating the pathological subtypes of CPs and their genetic mutational status.

**Figure 5:**
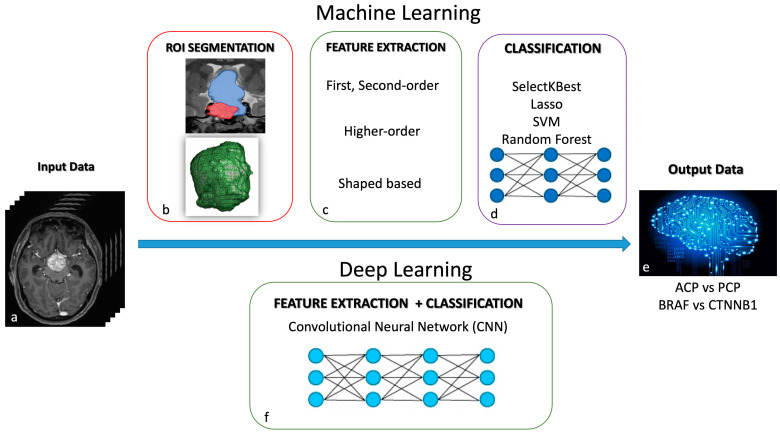
Radiomics in craniopharyngioma. Machine learning (ML) and deep learning (DL) techniques are AI subfields that use different diagnostic algorithms in quantitative bioimaging analysis. ML modeling (**a**–**e**): The computer receives inputs and features to create its own program for the desired output. Steps include ROI segmentation, feature extraction, feature selection (first- and second-order, higher-order, and shaped-based), and classification using models like the Random Forest, SelectKBest, least absolute shrinkage and selection operator (LASSO), and Support Vector Machine (SVM). DL modeling (**a**,**f**,**e**): It is a subset of ML, where the computer receives inputs and identifies features to generate the desired output. Convolutional neural networks (CNNs) learn from vast data to train a model.

Moreover, Ma et al. used a machine learning algorithm, the least absolute shrinkage and selection operator (LASSO), combining radiomics features with radiological characteristics (peritumoral edema) to build a model predicting preoperative CP invasiveness. These authors demonstrated that the radiomic–clinical nomogram had high predictive power for assessing the local invasiveness of ACPs [143]. These data contribute significantly to prognosis, as local invasion is closely associated with tumor adherence to surrounding tissues and the failure of complete resection, as suggested by several studies [105,134,144] (Figure 5).

### 4.6. Morbidity

Long-term morbidity is linked to tumor-related and/or treatment-related risk factors, including progressive disease with multiple recurrences, cerebrovascular disease, and chronic neuroendocrine deficiencies [145,146,147]. Insult to the hypothalamus is the most clinically relevant negative risk factor, having a profound impact on morbidity. This results in or exacerbates pre-existing disorders such as obesity, sleep disturbances, dysregulated temperature homeostasis, disruption to normal thirst and electrolyte regulation (including diabetes insipidus), as well as neurocognitive, psychosocial, and behavioral issues [28]. Major long-term neurocognitive complications include cognitive problems, particularly those affecting attention, working memory, episodic memory, and executive function. Psychosocial and behavioral issues result in difficulties in learning and emotional control, unsatisfactory peer relationships, as well as psychopathological symptoms such as anxiety, depression, and withdrawal [9]. It is reported that 35% of patients with craniopharyngiomas show symptoms and signs indicative of hypothalamic impairment at diagnosis, with this percentage increasing to between 65 and 80% after surgery or radiotherapy [148]. Visual disturbances represent another commonly observed long-term complication in both adults and children regardless of the chosen treatment method [28]. All of these factors translate into a reduction in quality of life in the short- and/or long-term and require a multidisciplinary care to manage and offer rehabilitation to those who are left with endocrine, metabolic, visual, neurocognitive, and psychosocial sequelae.

### 4.7. Role of the Multidisciplinary Team in the Management of the Craniopharingiomas

To achieve optimal outcomes in craniopharyngioma management, it is crucial to involve a multidisciplinary team of specialized neurosurgeons, neuroradiologists, neuro-oncologists, pathologists, and endocrinologists in diagnosis, surgery planning, irradiation, and long-term follow-up. Treatment strategies should be tailored on a case-by-case basis according to the patient’s age, tumor characteristics, size and location, and the patient’s overall condition. During the diagnostic phase, the imaging interpretation and the relationship between the tumor and adjacent structures, along with the evaluation of hypothalamic–pituitary axis dysfunction, guide the choice of treatment. Generally, if the tumor is favorably localized (with no anatomical involvement of the hypothalamic and optical structures), the therapy of choice is complete resection, meticulously performed to preserve hypothalamic and optic functions. On the other hand, a conservative approach is recommended for patients with hypothalamic involvement of craniopharyngioma at diagnosis. In these cases, the optimal therapy is limited hypothalamus-sparing radiotherapy, accompanied by partial resection, to minimize recurrences and progression [132,149,150]. In patients with mainly cystic tumor components, especially in children and young adults, intracavitary and interstitial treatment options should be considered [149,151]. The limited effectiveness and significant treatment-related complications of conventional therapies need the development of innovative approaches to improve the therapeutic outcomes and reduce the adverse effects. Currently, targeted therapies for craniopharyngiomas are gaining traction, aimed at specifically inhibiting the molecular pathways driving tumor growth, allowing for the reduction in treatment-related complications and preserving critical neurological and endocrine functions [5]. During follow-up, serial imaging is recommended to detect and treat potential complications related to treatment and disease progression. Regular endocrine evaluations to monitor and adjust hormone levels with appropriate replacement therapies are essential for managing the symptoms of hypothalamic–pituitary dysfunction, addressing growth and development issues, and implementing lifestyle modifications [19]. Additionally, psychological support is beneficial for managing obesity, sleep disorders, and other hypothalamic-related complications. This support helps in the recovery of cognitive and behavioral functions, thereby improving the patient’s quality of life [19,152].

## 5. Limitations

The primary limitation was the search method, limited to MEDLINE/PubMed, with studies published only in English being evaluated. Nonetheless, these selected studies may be considered the most representative to provide a coherent and comprehensive overview of the topic.

## 6. Conclusions

The management of CPs requires a multidisciplinary team to achieve better long-term results. Neuroradiologists, neurosurgeons, radiation oncologists, and endocrinologists need to collaborate closely to ensure the most appropriate tailored treatment decisions both in diagnosis and during follow-up.

Intraoperative CP topography is the major determinant of the degree of adherence to surrounding structures and, consequently, of the recurrence rate. However, preoperative imaging supported by radiomic quantitative analysis may allow for the accurate topographic localization and characterization of the tumor. Additionally, it may be useful for predicting the severity of hypothalamic–pituitary dysfunction and determining the most appropriate surgical strategy.

Early postsurgical imaging is crucial for assessing the presence and extent of residual disease, as well as for identifying potential complications arising from radiation therapy and surgical procedures. Imaging, endocrine evaluations, and psychological support are required for the identification and management of tumor recurrence to improve the patient’s quality of life in both the short- and long-term.

## Figures and Tables

**Table 1:** Differences between CP histotypes reported in the literature.

Category	Histotype	Author, Year	Findings Addressing Histotype Diagnosis
Mutation	PCP	Brastianos et al., 2016 [15]; Larkin et al., 2014 [14]	BRAF (V600E) mutation
	ACP	Preda et al., 2015 [11]	β-catenin (CTNNB1) gene mutation
Age	PCP	Brastianos et al., 2016 [15]; Müller et al., 2019 [9]; Bunin et al., 1998 [16]	More frequent in adults
	ACP	Müller et al., 2019 [9]; Zacharia et al., 2012 [6]; Nielsen et al., 2011 [12]	Bimodal distribution: children around 5–15 years, adults at 45–60 years (less common)
Topography	PCP	Pascual et al., 2013 [24]	Frequently strictly intraventricular CPs
	ACP	Pascual et al., 2013 [24]	Variable presence of sellar–suprasellar, pseudointraventricular, secondary intraventricular, and not-strictly intraventricular CPs
Radiological (MRI) appearance	PCP	Crotty et al., 1995 [25]; Pascual et al., 2013 [24]; Sartoretti-Schefer et al., 1997 [26]	Solid, rarely cystic; rounded morphology
	ACP	Gupta et al., 2006 [27]; Hamblin et al., 2021 [28]; Jipa et al., 2021 [29]; Karavitaki et al., 2014 [30]; Karavitaki et al., 2006 [2]; Sartoretti-Schefer et al., 1997 [26]	Predominantly cystic tumors (more common in the BRAF-WT tumors than in the BRAF-mutated tumors) or cystic tumors associated with a small solid component, bilobed or multilobulated appearance due to multiple cysts, and finger-shaped protrusions into the adjacent nervous tissue
Content of cysts	PCP	Mollá et al., 2002 [31]; Tariq et al., 2017 [32]; Karavitaki et al., 2006 [2]	Clear or yellow; rarely viscous
	ACP	Sartoretti-Schefer et al., 1997 [26]; Lithgow et al., 2000 [3]; Mollá et al., 2002 [31]	Machinery oil-like; rich in blood products and cholesterol clefts
Calcifications	PCP	Tariq et al., 2017 [32]; Crotty et al., 1995 [25]	Rare or absent
	ACP	Lee et al., 2016 [33]; Weiner et al., 1994 [34]; Adamson et al., 1990 [35]; Miller et al., 1994 [36]; Müller et al., 2019 [9]	ACPs characteristic; more common in pediatric population Eggshell-like calcifications along the cyst wall
Interface with adjacent tissue	PCP	Wu et al., 2022 [20]; Prieto et al., 2016 [37]	None
	ACP	Wu et al., 2022 [20]; Prieto et al., 2016 [37]; Higashi et al., 1990 [38]; Lee et al., 2016 [33]	Frequently glial reactive changes, brain invasion, or edema in infundibulo-tuberal, secondary intraventricular and pseudointraventricular CPs

ACPs, adamantinomatous craniopharyngiomas; CPCs, papillary craniopharyngiomas; CPs, craniopharyngiomas; MRI, magnetic resonance imaging; WT, wild type.

**Table 2:** Risk factors predictive of hypothalamic adherence and recurrence: summary of data from the literature.

**Category**	**Author, Year**	**Factors Predictive of Hypothalamic Adherence**
Histotype	Prieto et al., 2018 [39]	Mutations of the gene-encoding β-catenin (CTNNB1): higher expression of factors contributing to tight tumor adherence
Size	Katz et al., 1975 [40]; Shapiro et al., 1979 [41]; Sweet et al., 1980 [42]; Wen et al., 1989 [43]; Hetelekidis et al., 1993 [44]; Weiner et al., 1994 [34]; De Vile et al., 1996 [45]; Fahlbusch et al., 1999 [46]; Gupta et al., 2006 [27]; Shi et al., 2008 [47]; Elliott et al., 2010 [48]	Large size (3–5 cm): presenting tighter attachment to the surrounding neurovascular structures
Topography	Prieto et al., 2018 [39]; Prieto et al., 2016 [37]	Infundibulo-tuberal (or not-strictly intraventricular) and secondary intraventricular CPs: high adherence
Radiological (MRI) appearance	Prieto et al., 2016 [37]; Prieto et al., 2018 [39]; Higashi et al., 1990 [38]	Cystic appearance, multilobulated and dumb-bell tumor shape, and circumferential adherence patterns: high adherence
Contents of the cysts	Miller et al., 1994 [36]	Appearance of machinery oil: high adherence
Calcifications	Serbis et al., 2023 [49]; Adamson et al., 1990 [35]	Presence of calcifications as a marker of tight CP adhesions
Interface with adjacent tissue	Prieto et al., 2016 [37]; Higashi et al., 1990 [38]; Petito et al., 1996 [50]	Gliotic or inflammatory reaction of the adjacent brain tissue, edema-like changes as a marker of tight CP adhesions: predominantly in infundibulo-tuberal and secondary intraventricular CPs
**Category**	**Author, Year**	**Factors Predictive of Recurrence**
Size	Katz et al., 1975 [40]; Shapiro et al., 1979 [41]; Sweet et al., 1980 [42]; Wen et al., 1989 [43]; Hetelekidis et al., 1993 [44]; Weiner et al., 1994 [34]; De Vile et al., 1996 [45]; Fahlbusch et al., 1999 [46]; Gupta et al., 2006 [27]; Shi et al., 2008 [47]; Elliott et al., 2010 [48]	Large size (3–5 cm): total removal is more difficult
Topography	Fahlbusch et al., 1999 [46]; Shi et al., 2008 [47]; Van Effenterre et al., 2002 [51]; Prieto et al., 2017 [52]	Infundibulo-tuberal CPs and secondary intraventricular CPs: partial surgical removal due to their extensive attachments to the hypothalamus
Radiological (MRI) appearance	Katz et al., 1975 [40]; Metzger et al., 1979 [53]; Gupta et al., 2006 [27]	Cystic component: it is difficult to remove the capsule during surgery due to strong attachments to surrounding neurovascular structures, particularly if the capsule wall is thick or calcified
Contents of the cysts	Calandrelli et al., 2024 [54]	Viscous colloid cystic content: less extensive surgical excision and a higher likelihood of relapse during the follow-up period
Calcifications	Fahlbusch et al., 1999 [46]; Prieto et al., 2013 [55]; Fouda et al., 2021 [56]	Calcifications: incomplete surgical removal
Interface with adjacent tissue	Duff et al., 2000 [57]; Fahlbusch et al., 1999 [46]; Gupta et al., 2006 [27]; Yasargil et al., 1990 [58]	Loss of the peritumoral gliotic layer interposed between the CP and the surrounding hypothalamus after tumor resection: high likelihood of relapse during the follow-up period

CPs, craniopharyngiomas; MRI, magnetic resonance imaging.
